# Downregulation and forced expression of EWS-Fli1 fusion gene results in changes in the expression of G _1_ regulatory genes

**DOI:** 10.1054/bjoc.2000.1652

**Published:** 2001-03

**Authors:** Y Matsumoto, K Tanaka, F Nakatani, T Matsunobu, S Matsuda, Y Iwamoto

**Affiliations:** Department of Orthopaedic Surgery, Graduate School of Medical Sciences, Kyushu University, 3-1-1 Maidashi, Higashi-ku, Fukuoka, 812-8582, Japan

**Keywords:** EWS-Fli1, cell-cycle, G _0_/G _1_ arrest, antisense oligonucleotides, clinical samples, transfection

## Abstract

Chromosomal translocation t(11;22)(q24:q12) is detected in approximately 90% of tumours of the Ewing family (ET). This translocation results in EWS-Fli1 gene fusion which produces a EWS-Fli1 fusion protein acting as an aberrant transcriptional activator. We previously reported that the inhibition of EWS-Fli1 expression caused the G _0_/G _1_ arrest of ET cells. We, therefore, hypothesized that EWS-Fli1 may affect the expression of G _1_ regulatory genes. Downregulation of EWS-Fli1 fusion proteins was observed 48 hours after the treatment with EWS-Fli1 antisense oligonucleotides. The expressions of G _1_ cyclins, cyclin D1 and cyclin E, were markedly decreased in parallel with the reduction of EWS-Fli1 fusion protein. On the other hand, the expression of p21 and p27, which are important cyclin-dependent kinase inhibitors (CKIs) for G _1_–S transition, was dramatically increased after the treatment with EWS-Fli1 antisense oligonucleotides. RT-PCR analysis showed that alteration of the expressions of the cyclins and CKIs occurred at the mRNA level. Furthermore, transfection of EWS-Fli1 cDNA to NIH3T3 caused transformation of the cells and induction of the expression of cyclin D1 and E. Clinical samples of ET also showed a high level of expression of cyclin D1 mRNA, whereas mRNAs for p21 and p27 were not detected in the samples. These findings strongly suggest that the G _1_–S regulatory genes may be involved in downstream of EWS-Fli1 transcription factor, and that the unbalanced expression of G _1_–S regulatory factors caused by EWS-Fli1 may lead to the tumorigenesis of ET. © 2001 Cancer Research Campaign http://www. bjcancer.com


*British Journal of Cancer* (2001) **84**(6), 768–775
© 2001 Cancer Research Campaign
doi: 10.1054/ bjoc.2001.1652, available online at http://www.idealibrary.com on IDEAL®

### Downregulation and forced expression of EWS-Fli1 fusion gene results in changes in the expression of $G_1$ regulatory genes

**Y Matsumoto, K Tanaka, F Nakatani, T Matsunobu, S Matsuda and Y Iwamoto**

Department of Orthopaedic Surgery, Graduate School of Medical Sciences, Kyushu University, 3-1-1 Maidashi, Higashi-ku, Fukuoka 812-8582, Japan

**Summary** Chromosomal translocation t(11;22)(q24:q12) is detected in approximately 90% of tumours of the Ewing family (ET). This translocation results in EWS-Fli1 gene fusion which produces a EWS-Fli1 fusion protein acting as an aberrant transcriptional activator. We previously reported that the inhibition of EWS-Fli1 expression caused the $G_0/G_1$ arrest of ET cells. We, therefore, hypothesized that EWS-Fli1 may affect the expression of $G_1$ regulatory genes. Downregulation of EWS-Fli1 fusion proteins was observed 48 hours after the treatment with EWS-Fli1 antisense oligonucleotides. The expressions of $G_1$ cyclins, cyclin D1 and cyclin E, were markedly decreased in parallel with the reduction of EWS-Fli1 fusion protein. On the other hand, the expression of p21 and p27, which are important cyclin-dependent kinase inhibitors (CKIs) for $G_1$–S transition, was dramatically increased after the treatment with EWS-Fli1 antisense oligonucleotides. RT-PCR analysis showed that alteration of the expressions of the cyclins and CKIs occurred at the mRNA level. Furthermore, transfection of EWS-Fli1 cDNA to NIH3T3 caused transformation of the cells and induction of the expression of cyclin D1 and E. Clinical samples of ET also showed a high level of expression of cyclin D1 mRNA, whereas mRNAs for p21 and p27 were not detected in the samples. These findings strongly suggest that the $G_1$–S regulatory genes may be involved in downstream of EWS-Fli1 transcription factor, and that the unbalanced expression of $G_1$–S regulatory factors caused by EWS-Fli1 may lead to the tumorigenesis of ET. © 2001 Cancer Research Campaign http://www.bjcancer.com

**Keywords**: EWS-Fli1; cell-cycle; $G_0/G_1$ arrest; antisense oligonucleotides; clinical samples; transfection

Ewing family tumours (ET) including Ewing's sarcoma and PNET are common bone and soft-tissue tumours in adolescents and young adults. They are characterized by the presence of poorly differentiated round cells. The majority of ET has a reciprocal t(11; 22)(q24; q12) chromosomal translocation (Giovannini et al, 1994). Molecular analyses have shown that the rearrangement fuses the 5′-half of the EWS gene on chromosome 22 to the 3′-half of the Fli1 gene on chromosome 11. This chimaeric gene creates a fusion protein that has the biochemical characteristics of an aberrant transcription factor. NIH3T3 cells expressing the EWS-Fli1 protein formed colonies in soft-tissue agar and tumours in immunodeficient mice (May et al, 1993). Targeting the EWS-Fli1 fusion products of ET cells with antisense RNA results in a loss of tumorigenicity of ET cells (Ouchida et al, 1995). We previously reported that inhibition of the expression of EWS-Fli1 by the treatment with antisense oligonucleotides led to the growth arrest of various ET cell lines both in vitro and in vivo (Tanaka et al, 1997). These data demonstrated that continuous expression of fusion products may be needed for the growth of ET.

In EWS-Fli1 transfected cells, an mE2-C gene (murine cyclin-selective ubiquitin conjugase) was induced, suggesting that the biological effects of EWS-Fli1 could be, at least in part, mediated by transcriptional alteration of genes involved in cell-cycle regulation (Arvand et al, 1998). However, there has been no previous report indicating any correlation between EWS-Fli1 fusion product and the expression of cell-cycle regulatory genes. In mammalian cells, the major regulatory events leading to cell proliferation occur in the $G_1$-phase of the cell cycle. Since we have already found that the inhibition of the EWS-Fli1 fusion gene caused $G_0/G_1$ arrest (Tanaka et al, 1997), we hypothesized that the deranged expression of cyclins, cyclin-dependent kinases (CDKs), and/or CDK-inhibitors (CKIs) acting in $G_1$ may play critical roles in the growth and oncogenesis of ET.

In this study, the effects of EWS-Fli1 antisense oligonucleotides and forced expression of EWS-Fli1 cDNA on the expression of $G_1$ cyclins, CDKs and CKIs in ET cells were investigated. Downregulation and forced expression of the EWS-Fli1 fusion gene caused dramatic alteration of the expression of $G_1$–S regulatory genes. These data suggest that the $G_1$–S regulatory genes may be in the downstream of the EWS-Fli1 oncogenic transcription factor.

*Received 18 May 2000*
*Revised 20 November 2000*
*Accepted 30 November 2000*

*Correspondence to:* Y Iwamoto

### MATERIALS AND METHODS

### Materials

Antisense and sense phosphorothioate oligodeoxynucleotides purified by high performance liquid chromatography were purchased from Kurabo Biomedicals Co. (Osaka, Japan). The sequence of the antisense oligonucleotides was ATCCGT-GACGCCATTTTCTCTCCT (Tanaka et al, 1997) and the corresponding sense sequence was used as a control. Monoclonal antibodies to human cyclin D1, cyclin E, p21, p27, and polyclonal antibodies to mouse cyclin E and p21 were obtained from Santa Crutz Biotechnology (Santa Cruz, CA, USA).

### Cell lines and culture conditions

Human PNET cell line SK-N-MC and mouse fibroblast cell line NIH3T3 were obtained from the American Type Culture Collection (Rockville, MD, USA). PNKT-1, human PNET cells line, was established and characterized in our laboratory (Tanaka et al, 1995). The cells were maintained in Dulbecco's modified Eagle's medium (DMEM) (Nissui Pharmaceuticals Co., Tokyo, Japan) supplemented with 10% fetal bovine serum (FBS) for SK-N-MC and PNKT-1 or 10% calf serum (CS) for NIH3T3 and these cells were then incubated at 37˚C in a humidified atmosphere with 5% of $CO_2$ in the air.

### Cell growth assay

Cell growth assay was carried out as described (Tanaka et al, 1997). Briefly, various cell lines were seeded on 35-mm culture dishes at $10^5$ viable cells/dish with 1.5 ml of medium, and incubated at 37˚C. 24 h later, the media were replaced with media containing antisense or sense oligonucleotides, and the media supplemented with the oligomers were replaced every 24 h. After various time intervals, cell numbers were determined using a haemocytometer. Since the cell viability determined by the trypan blue exclusion test more than 95% throughout the experiments, the concentration of oligonucleotides were not toxic.

### Plasmids

EWS-Fli1 cDNA was isolated from a cDNA library of ET cell line, SK-N-MC, by PCR amplification using forward primer: 5′-GGCAAGCTTAGAGGGAGACGGACGTTGAGAGAACGAG-3, and reverse primer, 5′-GGCTCTAGAAGTAAGTGTGCAGG-ATAAGCATCAG-3′. The PCR product (~1.6 kb) was subcloned into the EcoRI and XbaI sites in the pcDNA3.1 (+) mammalian expression vector (Invitrogen, Groningen, Netherlands), giving the pcDNA3.1 (+)- EWS-Fli1 construct.

### Transfection of NIH3T3 cells

NIH3T3 cells were seeded in 6-well plates at a density of $0.5 \times 10^5$ cells/well and allowed to grow for overnight before transfection. The cells were washed twice with PBS and a mixture containing 4 μl of FuGENE6 (Behringer) and 1 μg of DNA (pcDNA3.1 (+) empty vector or pcDNA3.1(+)-EWS-Fli1) in 2 ml DMEM with 10% FBS was overlaid on the cells. Two days after the transfection, the cells were split in 1:15 dilution and incubated with 0.25 mg $ml^{-1}$ G418 (Gibco-BRL, Grand Island, NY, USA). The medium containing G418 was replaced every 3–4 days. Bulk population of G418-resistent clones was established after 14 days culture and was used for inoculation to nude mice.

### Tumorigenesis

The EWS-Fli1 transfected NIH3T3 cells ($1 \times 10^6$) were suspended in 0.1 ml DMEM with 10% FBS and inoculated subcutaneously into the back of the nude mice. Tumour growth was monitored and the established tumour mass was resected, minced and cultured to isolate the single clone.

### RNA extraction from cells and surgical specimens

The cells were plated at a density of $2 \times 10^4$ cells 2 $ml^{-1}$ of media in 35-mm dishes. The cells were incubated with antisense and sense oligonucleotides for various periods and total RNA was isolated from the cells using an RNA-easy kit (Quiagen, Hilden, Germany). ET tissues used for RNA isolation were frozen in liquid nitrogen immediately after the resection and total RNA was extracted using Isogen (Wako, Osaka, Japan)

### RT-PCR

Two μg of total RNA were subjected to reverse-transcription reaction using a Ready-to-Go cDNA synthesis kit (Amersham, Arlington Heights, IL, USA). Composition of the primers used for PCR has been described previously (Wang et al, 1996; Tanaka et al, 1997). PCR reactions were performed in a final volume of 50 μl for 30 cycles. Each PCR cycle consisted of a heat-denaturation step at 94˚C for 1 min, a primer annealing step at 55˚C for 30 s, and an extension step at 72˚C for 1 min. The PCR reaction was performed within the linear range of amplification determined by a preliminary study. The PCR products were analysed by 1.5% agarose gel (Sigma Co., St. Louis, MO, USA).

### Western blot analysis

Logarithmically growing cells ($2–4 \times 10^6$) at approximately 70% confluence were harvested and solubilized in an NP-40-based lysis buffer (20 mM Tris (pH 7.4), 250 mM NaCl, 1.0% NP40, 1 mM EDTA, 50 mg $ml^{-1}$ leupeptin, and 1 mM phenylmethylsulphonyl fluoride). After 10 min of incubation on ice, the cells were scraped, sonicated, and clarified by centrifugation at 14 000 rpm for 30 min at 4˚C. Protein quantitation was determined by a Bradford protein assay (Bio-Rad, Hercules, CA, USA). The samples were boiled for 5 min and 10 μg of total protein from each of the samples were run on a 4–12% gradient pre-cast MOPS-polyacrylamide gel (Novex, San Diego, CA, USA) and blotted onto a nitrocellulose filter. The filter was pre-treated with TBS containing 5% dry milk and 0.05% Triton X, for 2 h at room temperature. Then, the filter was incubated with the appropriate primary antibodies for 2 h at room temperature. After several washes, the horseradish peroxidase-conjugated secondary antibody (Biosource, Illinois, IL, USA) was added and the filter was then incubated at room temperature for 1 h. After a final wash, immunoreactivity of the blots was detected using an enhanced chemiluminescence system (Amersham). Data were analysed using an NIH image Ver 1.56.

### Immunofluorescence

Cells were grown on coverslips and cultured for 24 h in DMEM with 10% FBS or 10% CS, fixed for 10 min with 4% paraformaldehyde in phosphate-buffered saline (PBS), and permeabilized in 0.1% Triton-X100. Then, the specimens were treated for 1 h at room temperature with the appropriate primary antibodies. After washing with PBS, the specimens were incubated with a FITC conjugated anti-mouse or anti-rabbit IgG (Santa Crutz Biotechnology) for 45 min at room temperature. The samples were washed, mounted and examined by confocal laser scanning microscopy.

### Statistical analysis

All statistical analysis were carried out according to the student's *t*-test.

© 2001 Cancer Research Campaign

### RESULTS

### Effect of antisense oligonucleotides on the expression of EWS-Fli1 fusion protein

We synthesized and used the 25 mer antisense oligonucleotides corresponding to the 34 to 58 base positions of EWS-Fli1 fusion gene including the ATG initiation codon, as previously described (Tanaka et al, 1997). SK-N-MC cells treated with EWS-Fli1 antisense oligonucleotides were subjected to Western blot analysis to detect EWS-Fli1 protein. When the cells were incubated with antisense oligonucleotides for 12 h, the expression of EWS-Fli1 fusion protein was not significantly affected. However, after 24 h of cultivation, the expression of EWS-Fli1 protein was reduced to approximately 40% that of the control cells and only faint expression was observed after 48 h of incubation (Figure 1). RT-PCR analysis also showed similar expression pattern of EWS-Fli1 mRNA, as in Western blot analysis (data not shown).

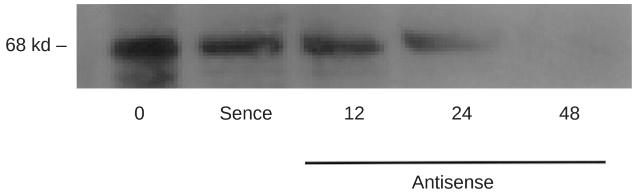

**Figure 1** Western blot analysis of EWS-Fli1 fusion protein in SK-N-MC cells. The cells were exposed for various periods to the sense or antisense oligonucleotides against EWS-Fli1 at a concentration of 10 µM. The cell lysate was extracted and subjected to Western blot analysis. A 68 kDa EWS-Fli1 fusion protein was detected. Treatment with sense oligonucleotides treatment did not affect the expression of EWS-Fli1 fusion protein, and the result at 48 h point is shown. The EWS-Fli1 protein expression was abolished at 48 h after the treatment

### Effect of antisense oligonucleotides on the expression of cyclin D1 and E

The $G_1$–S transition is mainly regulated by cyclin D, which binds to both CDK4 and CDK6, and by cyclin E, which binds to CDK2. We first investigated the effect of EWS-Fli1 antisense oligonucleotides on the expression of cyclin D1 and cyclin E in SK-N-MC cells, since it has been reported that cyclin D2 and cyclin D3 are not implicated in the development of various human tumours (Qian et al, 1998). Untreated SK-N-MC cells showed remarkable expression of cyclin D1 protein consistent with the findings of a recent report (Kovar et al, 1999). However, the expression level of cyclin D1 was reduced to 50% of that of the control after treatment with EWS-Fli1 antisense oligonucleotides for 48 h. The expression of cyclin E protein was slightly reduced after 24 h exposure with the antisense, and was reduced to approximately 10% of that of the control at 48 h point (Figure 2A). Treatment with sense oligonucleotides did not affect the expression of either of these cyclins at any time point. To further validate the results of Western blot analysis, protein distribution was analysed by immunofluorescence. As shown in Figure 2B, we observed marked nuclear staining of cyclin E in control SK-N-MC cells, however, incubation

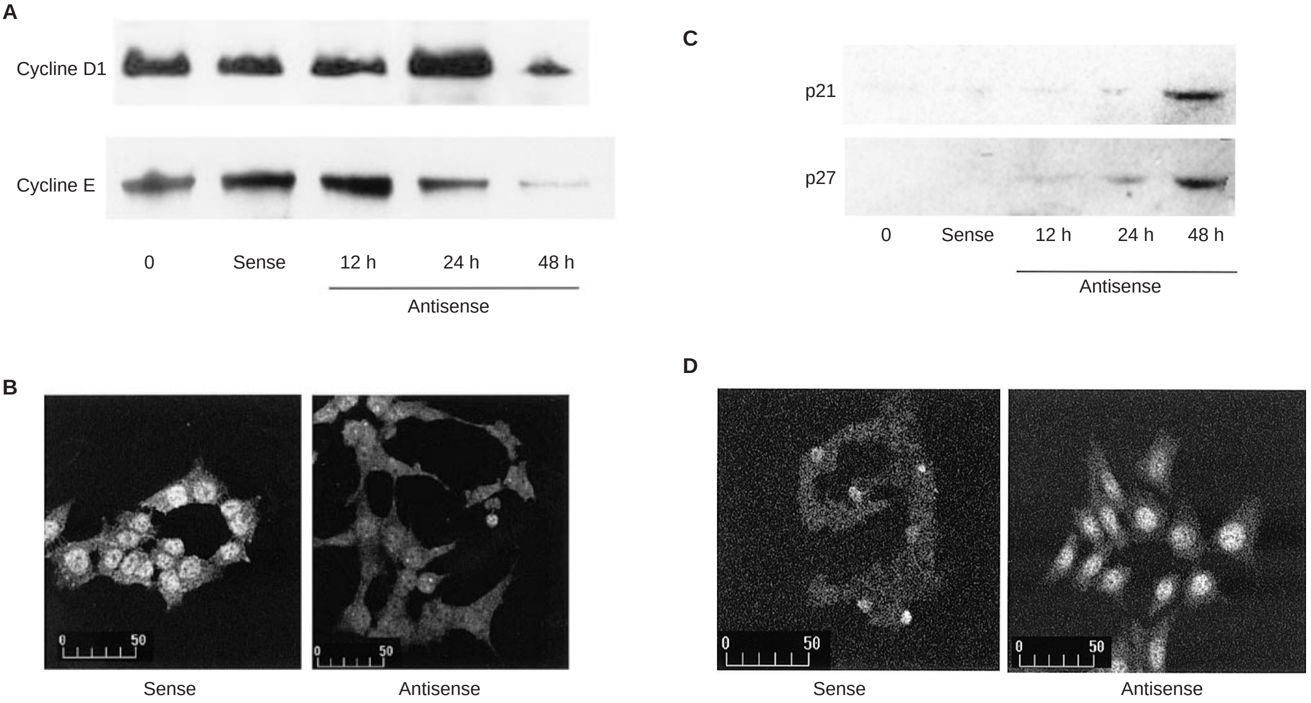

**Figure 2** Expression of cyclin D1, cyclin E, p21 and p27 proteins in SK-N-MC cells treated with antisense oligonucleotides. Total proteins were obtained at the indicated time after the addition of antisense or sense oligonucleotides. **(A)** Western blot analysis showed that the expressions of cyclin D1 and cyclin E were diminished by treatment with antisense oligonucleotides for 48 h, whereas the treatment with sense oligonucleotides did not affect the expressions.
**(B)** Immunofluorescence analysis of SK-N-MC cells using anti-cyclin E antibody. The cells incubated for 48 h with sense oligonucleotides showed strong nuclear staining of cyclin E, whereas almost no staining was observed after antisense exposure for 48 h. Bar, 50 µm. **(C)** Western blot analysis revealed that the expression of p21 and p27 proteins were induced by treatment with antisense oligonucleotides for 48 h. **(D)** In the immunofluorescence study, remarkable p21 expression within the nucleus was detected after antisense exposure for 48 h, while a very low-level expression of p21 was observed in sense-treated cells. Bar, 50 µm

*© 2001 Cancer Research Campaign*

with the EWS-Fli1 antisense oligonucleotides for 48 h totally abolished the nuclear stainings of cyclin E. Immunofluorescence analysis of cyclin D1 showed a similar staining pattern to that of cyclin E (data not shown).

### Effect of antisense oligonucleotides on the expression of CKIs associated with $G_I$–S transition

It has been shown that the cyclin-Cdk complex is inactivated by an excess of CKIs (Sherr and Roberts, 1995). Thus, we examined whether the protein expression of CKIs could also be altered by the treatment of SK-N-MC cells with antisense oligonucleotides. Western blot analysis showed almost no signal of p21 protein in untreated SK-N-MC cells. However, treatment with 10 μM antisense oligonucleotides for 24 h induced p21 expression, and a strong induction of p21 was observed at 48 h after the addition of the antisense. We also investigated the induction of another CKI, p27, in SK-N-MC cells. The p27 protein was induced in a time-dependent manner by the antisense treatment, whereas untreated and sense-treated SK-N-MC cells exhibited no expression of p27 protein (Figure 2C). Consistent with the results of Western blot analysis, p21 protein was detected by immunohistochemistry in SK-N-MC cells treated with the antisense oligonucleotides (Figure 2D). The other important CKI, p16, is frequently mutated in soft-tissue sarcomas. However, SK-N-MC cells were revealed to have intact p16 (Kovar et al, 1997), and treated with EWS-Fli1 antisense oligonucleotides did not affect p16 expression (data not shown). Therefore, the induction of p21 and p27 proteins may lead to inactivation of the cyclin-Cdk complex in antisense-treated SK-N-MC cells.

To investigate the effect of EWS-Fli1 antisense oligonucleotides on other ET cell lines, we treated PNKT-1, a well-characterized PNET cells expressing EWS-Fli1 fusion protein (Tanaka et al, 1997). $G_0/G_1$ growth arrest with the induction of p21 and p27 was also seen in the PNKT-1 cells treated with antisense oligonucleotides (data not shown). These results also suggest that there is a correlation between the downregulation of EWS-Fli1 fusion protein and the alteration of the expression of cell-cycle regulatory proteins.

### The antisense effect on mRNA expression of $G_1$ cyclins and CKIs

To examine the mechanism by which the antisense oligonucleotides modified the protein expression of cyclins and CKIs, we investigated the alteration of mRNA expression by RT-PCR analysis. Cyclin D1 and cyclin E mRNA levels in SK-N-MC cells were dramatically decreased by the treatment with antisense oligonucleotides for 36 h (Figure 3A). With regard to CKIs, the p21 mRNA level was increased in SK-N-MC cells following antisense treatment for 24 h and continued to increase for 48 h. However, treatment with antisense oligonucleotides for 12 h was not sufficient to produce upregulation of p21 mRNA expression. An increase in the p27 mRNA expression level was observed when the cells were treated with antisense for 48 h. The treatment with sense oligonucleotides had no effect on the mRNA levels of the cyclins and CKIs even after 48 h of treatment (Figure 3B). These data suggest that modification of the protein expressions of the cyclins and CKIs was mediated via induction of mRNAs or a reduction in the levels of mRNAs brought about by the challenge of the antisense oligonucleotides. Expressions of the mRNAs of p16, and CDK2, 4 and 6 were not significantly affected by the antisense treatment (data not shown).

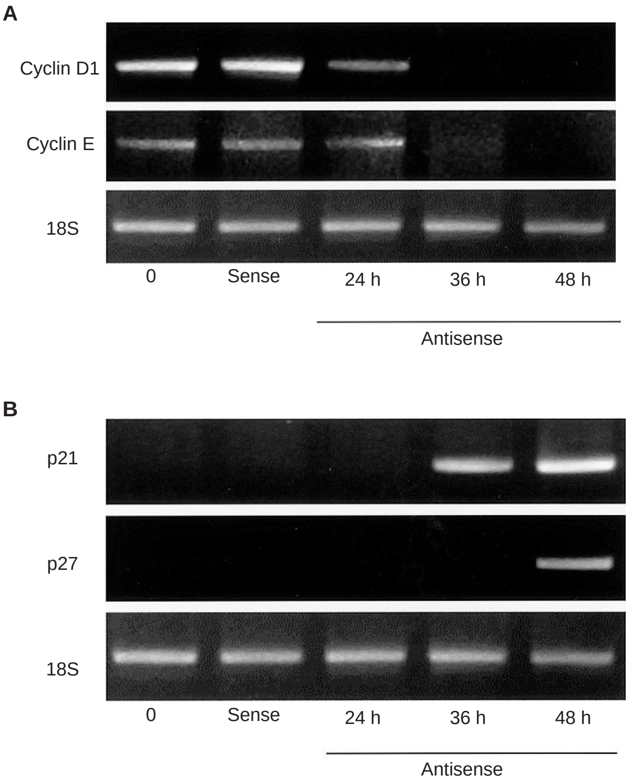

**Figure 3** Effect of antisense oligonucleotide on mRNA expression of cyclin D1, cyclin E **(A)** p21 and p27 **(B)** in SK-N-MC cells. Two μg of total RNA from the cells were reverse transcribed and cDNAs were subjected to PCR. The PCR products were electrophoresed on a 1.5% agarose gel. Ribosomal 18S RNA amplification served as an internal control. The results of mRNA expression of the cells treated with sense oligonucleotides for 48 h were also demonstrated. Consistent with the data shown in Figure 2, the antisense treatment inhibited mRNA expression of cyclin D1 and cyclin E, and induced p21 and p27 expression

### Establishment of the EWS-Fli1 transfected NIH3T3 cells, NIH3T3/EWS-Fli1

To investigate the effect of forced expression of the EWS-Fli1 fusion gene in non-transformed cells, we first tansfected the EWS-Fli1 cDNA into NIH3T3 cells. The transfected cells were selected by G418 treatment and bulk population of the transfected cells was inoculated into nude mice. Transfected cells developed tumour mass about 3 weeks after the inoculation. The tumour mass was resected, minced and plated on culture dishes. Then, a single clone, NIH3T3/EWS-Fli1 was isolated and established. The expression of EWS-Fli1 in NIH3T3/EWS-Fli1 was confirmed by RT-PCR analysis (Figure 4A). NIH3T3/EWS-Fli1 cells showed significantly rapid growth compared to its parental cells (Figure 4B). When NIH3T3/EWS-Fli1 cells were treated with antisense oligonucleotide, the growth of the cells was significantly inhibited; the growth inhibition rate was approximately 30% (Figure 4C). None of the oligonucleotides affected the growth of NIH3T3 cells (data not shown).

### Expression of $G_1$ cyclins and CKIs in the NIH3T3/EWS-Fli1 cells

As shown in Figure 5A, nuclear stainings of cyclin D1 and cyclin E were detected by immunofluorescence immunohistochemistry,

© 2001 Cancer Research Campaign 

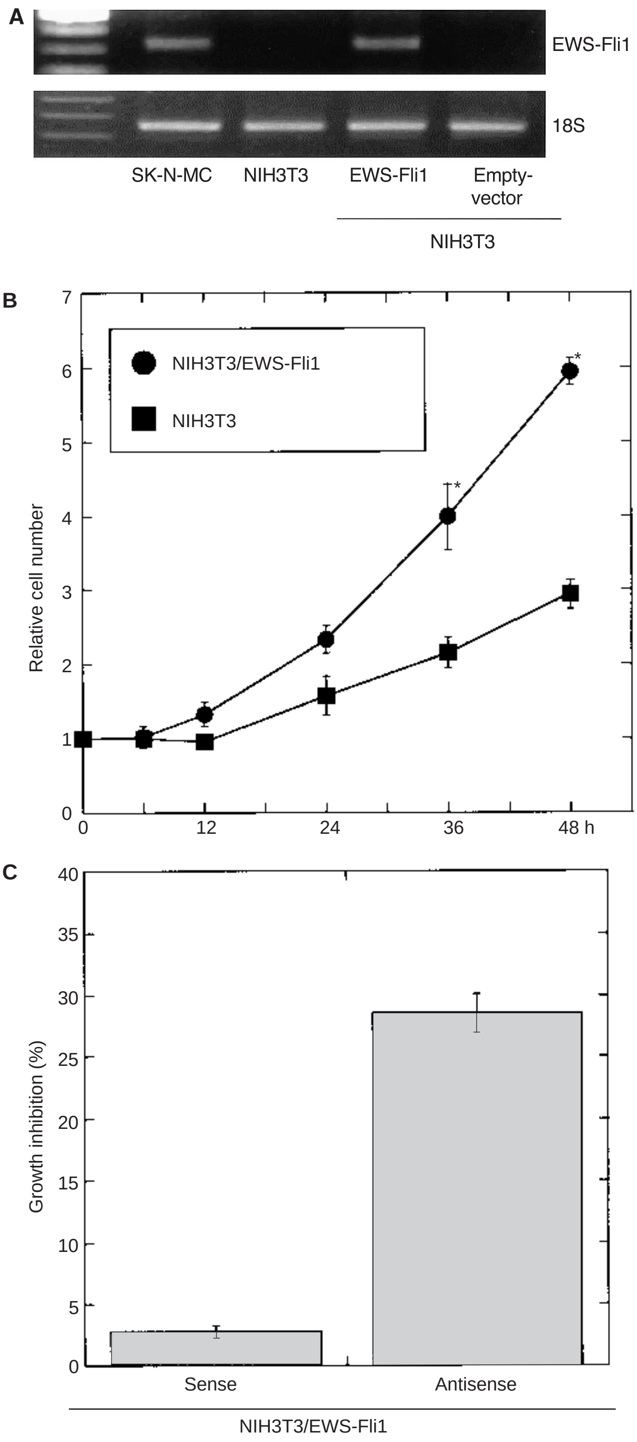

**Figure 4** Expression of EWS-Fli1 fusion gene and growth of EWS-Fli1 transfected NIH3T3 cells (NIH3T3/EWS-Fli1). **(A)** Demonstration of the presence of mRNA of EWS-Fli1 fusion gene in NIH3T3/EWS-Fli1. Total RNA from the SK-N-MC cells were used as the positive control for EWS-Fli1 expression. NIH3T3/EWS-Fli1 cells indicated clear expression of EWS-Fli1 fusion gene. Ribosomal 18S RNA amplification served as an internal control. **(B)** NIH3T3 and NIH3T3/EWS-Fli1 were plated at a concentration of $5 \times 10^4$ cells in 35 mm dishes. After various time intervals, the cell number was counted. Points and bars represent mean value and S.D. respectively. Significantly different from the parent NIH3T3 cells ($P < 0.01$). All experiments were carried out in triplicate and repeated twice. **(C)** Effect of sense and antisense oligonucleotides on NIH3T3/EWS-Fli1 cell proliferation. The cells were incubated with 10 μM sense or antisense oligonucleotides and the number of viable cells was counted after a 36-h incubation. The data represent the means of the 3 experiments. The bars represent S.D

however no expressions of both cyclins were observed in parental and empty-vector transfected cells. Consistent with the results of immunohistochemistry, Western blotting analysis indicated the strong expressions of cyclin D1 and cyclin E in NIH3T3/EWS-Fli1 (Figure 5B).

### Expression of $G_1$ cyclins and CKIs in the surgical specimens of ET

To investigate the expression levels of $G_1$ cyclins and CKIs in surgical specimens, total RNA was prepared from 4 samples of resected ET tissues. Reverse-transcribed cDNA from the tissues was subjected to PCR analysis. All samples showed EWS-Fli1 fusion gene expression (data not shown). As shown in Figure 6, the tissue specimens strongly expressed cyclin D1 mRNA. Cyclin E mRNA expression, which was observed in the SK-N-MC cells, was not detected in the tissues. No samples showed expression of p21 or p27 mRNA. These observations suggest that an imbalance

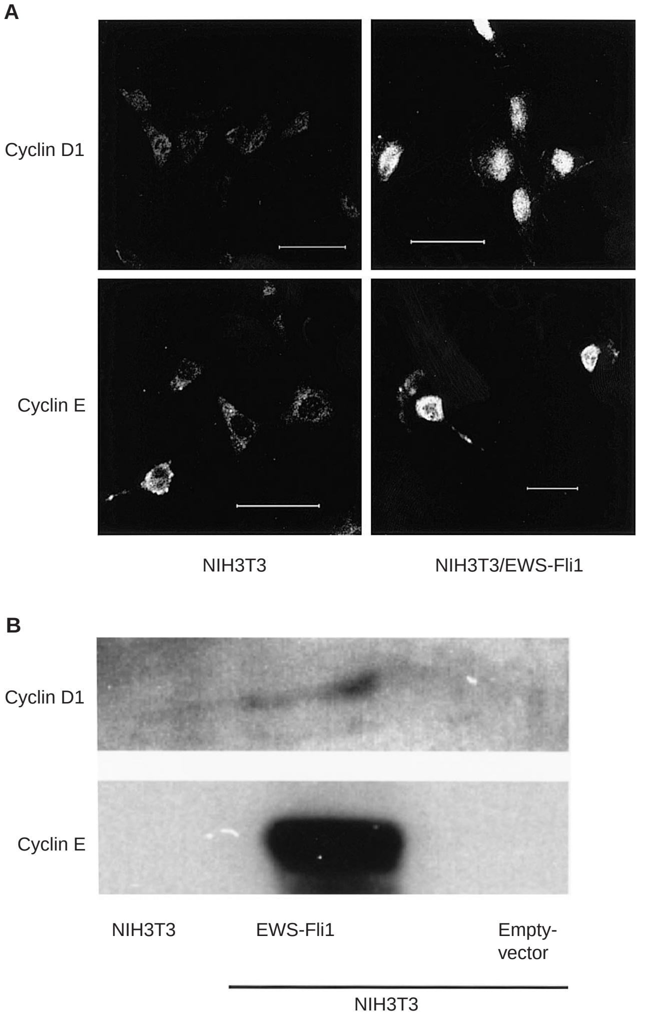

**Figure 5** Expression of cyclin D1 and cyclin E in NIH3T3/EWS-Fli1 cells. **(A)** Immunofluorescence analysis of NIH3T3/EWS-Fli1 using anti-cyclin D1 and cyclin E antibodies. The NIH3T3/EWS-Fli1 showed strong nuclear staining of both cyclins, whereas almost no staining was observed in NIH3T3 cells. Bar, 50 μm. **(B)** Western blot analysis of cyclin D1 and cyclin E expression. The expressions of cyclin D1 and cyclin E were induced by the trasfection of EWS-Fli1 fusion gene to NIH3T3 cells. Transfection of the pcDNA3.1(+) empty-vector did not affect the expressions of both cyclins

© 2001 Cancer Research Campaign

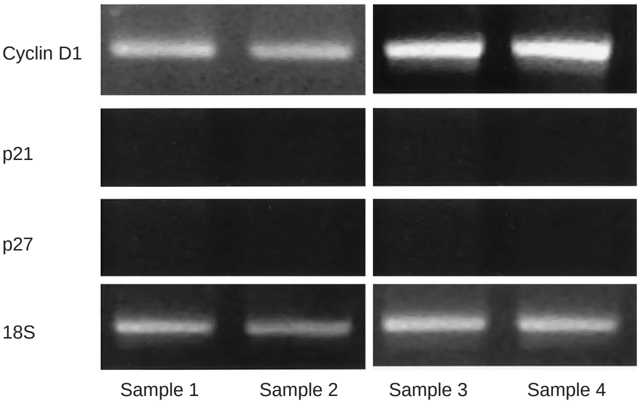

**Figure 6** RT-PCR analysis of cyclin D1, and p21 and p27 mRNA expression in biopsy samples of Ewing's sarcoma. Two μg of total RNA from biopsy samples were reverse transcribed. The cDNAs were subjected to PCR and electrophoresed on a 1.5% agarose gel. All surgical specimens showed mRNA expression of cyclin D1; whereas no mRNA expression of p21 and p27 was observed between $G_1$ cyclin and CKIs exists not only in ET cell lines and EWS-Fli1 fusion gene transfected cells but also in clinical ET samples.

### DISCUSSION

Malignant tumours are thought to be a disease with uncontrolled cell proliferation. There is a growing list of oncogene/tumour suppressor gene products that regulate the cell cycle, including c-myc, p53 and RB (Bedi et al, 1994; McGahon et al, 1994). From the results of our previous study, EWS-Fli1 fusion product is considered to be involved in cell-cycle progression, especially in $G_1$–S transition (Tanaka et al, 1997). In this study, we were able to show that alteration of the expression of $G_1$ regulatory factors caused by EWS-Fli1 antisense oligonucleotides results in the $G_0/G_1$ arrest of ET cells, and that exogenous transfection of EWS-Fli1 fusion gene induced the expressions of cyclin D1 and E in transformed NIH3T3 cells.

Cell-cycle progression is controlled by a complex of cyclins and CDKs counterbalanced by CKIs. Cyclin D1 binds and activates CDK4 (or CDK6). The cyclin D1-CDK4 complex phosphorylates retinoblastoma protein (pRB) and facilitates cell-cycle progression from the $G_1$ phase to the S phase. Forced expression of cyclin D1 in rodent mammary cells caused transformation both in vivo and in vitro (Wang et al, 1994). Cyclin E, a regulatory subunit of CDK2, is also known to be a factor controlling the $G_1$–S transition. According to the previous reports, the expression of cyclin E was highly enhanced in cell lines and surgical specimens of breast cancer (Keyomarsi et al, 1995). On the other hand, two gene families of mammalian CKIs have been identified: one family includes p16 (INK4A), p15 (INK4B) and p18 and the other includes p21Cip, p27Kip and p57. It has been reported that p21, p27 and p57 inhibit the kinase activity of cyclins D-CDK4 and 6 and the cyclin E-CDK2 complex in vitro (Gu et al, 1993). Overexpression of p21 and p27 keeps the RB protein in its active and hypophosphorylated form, and causes $G_0/G_1$ arrest. In our study, SK-N-MC cells showed a high steady expression of cyclins D1 and E both at the mRNA and protein levels. However, the mRNA and protein expressions of p21 and p27 in the cells were not observed. Surgical specimens also showed a high level of the expression of cyclin D1, although p21 and p27 expressions were not detected in the tissues. Furthermore, forced expression of EWS-Fli1 fusion gene caused transformation of NIH3T3 cells and the transformed cells showed strong expressions of cyclin D1 and cyclin E compared with the parental cells. Since the proper ratio of CDK-cyclin complex to CKIs is critical for $G_1$ progression, our observations suggest that an imbalance between the $G_1$ cyclin-CDK complex and p21 and/or p27 in ET cells and EWS-Fli1 fusion gene transfected cells may be the reason for uncontrolled proliferation, leading to their transformation.

We used antisense oligonucleotides against EWS-Fli1 targeting the sequence including the AUG initiation codon. These antisense oligonucleotides exert their effect by inhibiting the expression of the EWS-Fli1 gene. We have already demonstrated that the antisense oligonucleotides suppressed not only EWS-Fli1, but also endogenous EWS mRNA expression (Tanaka et al, 1997). However, the antisense oligonucleotides did not affect the growth and expression of cyclins and CKIs in other cell lines without the fusion gene, such as NIH3T3, IMR32 and IMR90 (data not shown). Therefore, we concluded that the effects of the antisense oligonucleotides were mainly mediated by the suppression of EWS-Fli1 expression.

The effect of antisense oligonucleotides on EWS-Fli1 appeared when the cells were exposed to antisense oligonucleotides for more than 24 h. The inhibition of EWS-Fli1 expression was followed by changes in the expression of cyclins and CKIs. Treatment of SK-N-MC cells with the antisense oligonucleotides reduced the mRNA and protein expression of cyclin D1. Coupling reduction of cyclin E mRNA and protein levels was also observed after the antisense treatment. The data indicate that the reduction of cyclin D1 and E by antisense oligonucleotides was due to a downregulation of mRNA expression. Transfection of EWS-Fli1 fusion gene in NIH3T3 cells induced the expression of cyclin D1 and cyclin E. It has been reported that an ets binding consensus sequence exists in the promoter region of cyclin D (Albanese et al, 1995), Since EWS-Fli1 act as a transcriptional factor via ets domain in the Fli1 portion, EWS-Fli1 may transactivate cyclin D1 expression through the ets binding sequence in the promoter. On the other hand, no ets binding sites have been indentified in the promoter region of cyclin E gene. Recent studies revealed that the abundance of cyclin E protein was controlled at transcriptional level (Botz et al, 1996) and/or stability of mRNA (Oda et al, 1995). Thus, it is possible that induction of cyclin E expression by EWS-Fli1 may be due to the stabilization of mRNA of cyclin E. However, exact mechanism of the EWS-Fli1 regulation of $G_1$ cyclins need to be further investigated.

We also investigated the phosphorylated RB protein (ppRB) expression in the untreated SK-N-MC cells. Recent study revealed that the SK-N-MC cells showed only weak expression of ppRB (Kovar et al, 1997). However, in our study, the expression of ppRB was observed in untreated and EWS-Fli1 sense oligonucleotides treated SK-N-MC cells. In addition, treatment with the EWS-Fli1 antisense oligonucleotides caused the reduction of the expression level of ppRB (data not shown). These findings suggest that the reduction of the expression level of ppRB might be caused by the alteration of $G_1$ regulatory genes induced by EWS-Fli1 antisense treatment. In regard to the ppRB expression of the NIH3T3/EWS-Fli1 cells, transfection of EWS-Fli1 cDNA induced phosphorylated forms of pRB (data not shown) in NIH3T3 cells, which may be the result of the induction of cyclin D1 and E in these cells.

*© 2001 Cancer Research Campaign* 

The antisense-treated cells expressed both p21 and p27 to a remarkable degree, whereas sense-treated cells did not. That induction occurred in a time-dependent manner both at the mRNA and protein levels. In our results, logarithmically growing NIH3T3 cells did not show the expressions of p21 and p27. Thus, the inhibitory effect of the transfection of EWS-Fli1 fusion gene on the expressions of p21 and p27 could not be confirmed in this study. It is well known that the p21 gene is transcriptionally activated by wild-type p53 protein (el-Deiry et al, 1998), suggesting that p21 plays a key role as a downstream mediator of p53-induced cell growth arrest. Although surgical specimens of ET usually reveal to express wild-type p53, it has been reported that the p53 gene is mutated in SK-N-MC cells (Qian et al, 1998). Several reports have demonstrated the p53-independent regulation of p21 gene expression. Transcription factors, such as STAT protein (Chin et al, 1996) or vitamin D3 receptor (Liu et al, 1996), act via direct specific binding to a promoter in p21 gene. In human prostate carcinoma cell lines lacking wild-type p53, Lovastatim, an inhibitor of HMG-CoA reductase, transactivated p21 gene expression through an SP-1-binding site in the promoter and induced growth arrest in the $G_1$ phase (Lee et al, 1998). Furthermore, a recent report indicated that the ets-binding sites were located in the promoter region of p21 (Funaoka et al, 1997). We investigated the effect of the antisense oligonucleotides on transactivation of p21 by luciferase assay using the p21 promoter luciferase reporter construct. When SK-N-MC cells were treated with antisense oligo, 2 to 3-fold induction of the reporter gene activity was observed (data not shown). These data suggest that EWS-Fli1 may be a key regulatory factor for expression of p21 in ET cells.

It has been previously demonstrated that the abundance of p27 is regulated by the ubiquitin-proteasome pathway (Pagano et al, 1995), and that the expression level of p27 mRNA remained unchanged in most cases. Our results indicated, however, that the administration of EWS-Fli1 antisense oligonucleotides clearly increased the p27 mRNA and protein expression. The previous study showed that cAMP-analogue can induce the mRNA and protein expression of p27 in T lymphocytes (Lalli et al, 1996), and it also caused cell-cycle block in the $G_1$ phase in CHP-100 Ewing's sarcoma cells (Srivastava et al, 1998). In the present study, the amount of intracellular cAMP was not changed after the treatment with the antisense oligonucleotides, indicating that the cAMP-pathway is perhaps not involved downstream of EWS-Fli1 (data not shown).

In conclusion, our results demonstrate that suppression of the EWS-Fli1 fusion protein by the antisense oligonucleotides altered the expression of $G_1$ regulatory cyclins and CKIs, thereby resulting in the $G_1$ growth arrest in ET cell lines. Enforced expression of EWS-Fli1 resulted in transformation of NIH3T3 cells, and strong induction of cyclin D1 and cyclin E was observed in the transformed cells. These data suggest that EWS-Fli1 may affect the $G_1$– S transition in ET cells and that $G_1$ cyclins and CKIs may be the direct or indirect target of EWS-Fli1 fusion transcription factor.

### ACKNOWLEDGEMENT

This study was supported in part by a Grant-in-Aid for Scientific Research (12557125 and 10307034) from Japan Society for the Promotion of Science, and a Grant-in-Aid for Cancer Research from the Ministry of Health and Welfare, Japan.

### REFERENCES

Albanese C, Johnson J, Watanabe G, Eklund N, Vu D, Arnold A and Pestell G (1995) Transforming p21ras mutants and c-Ets-2 activate the cyclin D1 promoter through distinguishable regions, *J Biol Chem* **270**: 23589–23597

Arvand A, Bastians H, Welford SM, Thompson AD, Ruderman JV and Denny CT, (1998) EWS/Fli1 up-regulates mE2-C, a cyclin-selective ubiquitin conjugating enzyme involved in cyclin B destruction. *Oncogene* **17**: 2039–2045

Bedi A, Zehnbauer BA, Barber JP, Sharkis SJ and Jones RJ (1994) Inhibition of apoptosis by BCR-ABL in chronic myeloid leukemia. *Blood* **83**: 2038–2044

Botz, Zerfass-Thome K, Spitokovsky D, Deilus H, Vogt B, Eilers M, Hatzigeorgiou A and Jansen-Durr P (1996) Cell cycle regulation of the murine cyclin E gene depends on an E2F binding site in the promoter. *Mol Cell Biol* **16**: 3401–3409

Chin YE, Kitagawa M, Su WC, You ZH, Iwamoto Y and Fu XY (1996) Cell growth arrest and induction of cyclin-dependent kinase inhibitor p21 WAF1/CIP1 mediated by STAT1. *Science* **272**: 719–722

el-Deiry WS, Tokino T, Velculescu VE, Levy DB, Parsons R, Trent JM, Lin D, Mercer WE, Kinzler KW and Vogelstein B (1993) WAF1, a potential mediator of p53 tumor suppression. *Cell* **75**: 817–825

Funaoka K, Shindoh M, Yoshida K, Hanazawa M, Hida K, Nishikata S, Totsuka Y and Fujinaga K (1997) Activation of the p21 (Waf1/Cip1) promoter by ets oncogene family transcription factor E1AF. *Biochem Biophys Res Commun* **236**: 79–82

Geng Y, Whoriskey W, Park MY, Bronson RT, Medema RH, Li T, Weinberg RA and Sicinski P (1999) Rescue of cyclin D1 deficiency by knock in cyclin *E Cell* **97**: 767–777

Giovannini M, Biegel JA, Serra M, Wang JY, Wei YH, Nycum L, Emanuel BS and Evans GA (1994) EWS-erg and EWS-Fli1 fusion transcripts in Ewing's sarcoma and primitive neuroectodermal tumors with variant translocations. *J Clin Invest* **94**: 489–496

Gu Y, Turck CW and Morgan DO (1993) Inhibition of CDK2 activity in vivo by an associated 20K regulatory subunit. *Nature* **366**: 707–710

Keyomarsi K, Conte D Jr. Toyofuku W and Fox MP (1995) Deregulation of cyclin E in breast cancer. *Oncogene* **11**: 941–950

Kovar H, Jug G, Aryee DN, Zoubek A, Ambros P, Gruber B, Windhager R and Gadner H (1997) Among genes involved in the RB dependent cell cycle regulatory cascade, the p16 tumor suppressor gene is frequently lost in the Ewing family of tumors. *Oncogene* **15**: 2225–2232

Lalli E, Sassone-Corsi P and Ceredig R (1996) Block of T lymphocyte differentiation by activation of the cAMP-dependent signal transduction pathway. *EMBO J* **15**: 528–537

Lee SJ, Ha MJ, Lee J, Nguyen P, Choi YH, Pirnia F, Kang WK, Wang XF, Kim SJ and Trepel JB (1998) Inhibition of the 3-hydroxy-3-methylglutaryl-coenzyme A reductase pathway induces p53-independent transcriptional regulation of p21(WAF1/CIP1) in human prostate carcinoma cells. *J Biol Chem* **273**: 10618–10623

Liu M, Lee MH, Cohen M, Bommakanti M and Freedman LP (1996) Transcriptional activation of the Cdk inhibitor p21 by vitamin D3 leads to the induced differentiation of the myelomonocytic cell line U937. *Genes Dev* **10**: 142–153

Lukas J, Herzinger T, Hansen K, Moroni MC, Resnitzky D, Helin K, Reed SI and Bartek J (1997) Cyclin E-induced S phase without activation of the pRb/E2F pathway. *Genes Dev* **11**: 1479–1492

May WA, Gishizky ML, Lessnick SL, Lunsford LB, Lewis BC, Delattre O, Zucman J, Thomas G and Denny CT (1993) Ewing sarcoma 11;22 translocation produces a chimeric transcription factor that requires the DNA-binding domain encoded by FLI1 for transformation. *Proc Natl Acad Sci USA* **90**: 5752–5756

McGahon A, Bissonnette R, Schmitt M, Cotter KM, Green DR and Cotter TG (1994) BCR-ABL maintains resistance of chronic myelogenous leukemia cells to apoptotic cell death [published erratum, appears in Blood 1994 Jun 15;83(12):3835]. *Blood* **83**: 1179–1187

Ouchida M, Ohno T, Fujimura Y, Rao VN and Reddy ES (1995) Loss of tumorigenicity of Ewing's sarcoma cells expressing antisense RNA to EWS-fusion transcripts. *Oncogene* **11**: 1049–1054

Pagano M, Tam SW, Theodoras AM, Beer-Romero P, Del Sal G, Chau V, Yew PR, Draetta GF and Rolfe M (1995) Role of the ubiquitin-proteasome pathway in regulating abundance of the cyclin-dependent kinase inhibitor p27 [see comments]. *Science* **269**: 682–685

Qian X, Kulig E, Jin L and Lloyd RV (1998) Expression of D-type cyclins in normal and neoplastic rat pituitary. *Endocrinology* **139**: 2058–2067

Sherr CJ and Roberts JM (1995) Inhibitors of mammalian G1 cyclin-dependent kinases. [Review] [153 refs] *Genes Dev* **9**: 1149–1163

 © 2001 Cancer Research Campaign

Srivastava RK, Srivastava AR and Cho-Chung YS (1998) Synergistic effects of 8-chlorocyclic-AMP and retinoic acid on induction of apoptosis in Ewing's sarcoma CHP-100 cells. *Clin Cancer Res* **4**: 755–761
Tanaka K, Iwamoto Y, Noguchi Y, Oda Y and Sugioka Y (1995) The establishment and characterization of a peripheral neuroepithelioma cell line in soft tissue of extremity. *Lab Invest* **72**: 237–248
Tanaka K, Iwakuma T, Harimaya K, Sato H and Iwamoto Y (1997) EWS-Fli1 antisense oligodeoxynucleotide inhibits proliferation of human Ewing's sarcoma and primitive neuroectodermal tumor cells. *J Clin Invest* **99**: 239–247
Waga S, Hannon GJ, Beach D and Stillman B (1994) The p21 inhibitor of cyclin-dependent kinases controls DNA replication by interaction with PCNA [see comments]. *Nature* **369**: 574–578
Wang TC, Cardiff RD, Zukerberg L, Lees E, Arnold A and Schmidt EV (1994) Mammary hyperplasia and carcinoma in MMTV-cyclin D1 transgenic mice. *Nature* **369**: 669–671
Wong H and Riabowol K (1996) Differential CDK-inhibitor gene expression in aging human diploid fibroblasts. *Exp Gerontol* **31**: 311–325

*© 2001 Cancer Research Campaign*
